# Multi-Camera-Based Universal Measurement Method for 6-DOF of Rigid Bodies in World Coordinate System

**DOI:** 10.3390/s20195547

**Published:** 2020-09-28

**Authors:** Zuoxi Zhao, Yuchang Zhu, Yuanhong Li, Zhi Qiu, Yangfan Luo, Chaoshi Xie, Zhuangzhuang Zhang

**Affiliations:** 1 College of Engineering, South China Agricultural University, Guangzhou 510642, China; zhu_yuchang@stu.scau.edu.cn (Y.Z.); liyuanhong@stu.scau.edu.cn (Y.L.); qiuzhi@stu.scau.edu.cn (Z.Q.); luoyangfan@stu.scau.edu.cn (Y.L.); xxcs@stu.scau.edu.cn (C.X.); zzz@stu.scau.edu.cn (Z.Z.); 2 Key Laboratory of Key Technology on Agricultural Machine and Equipment (South China Agricultural University), Ministry of Education, Guangzhou 510642, China

**Keywords:** rigid body, six-degrees-of-freedom measurement, multiple cameras, Zhang Zhengyou’s calibration, world coordinate system

## Abstract

The measurement of six-degrees-of-freedom (6-DOF) of rigid bodies plays an important role in many industries, but it often requires the use of professional instruments and software, or has limitations on the shape of measured objects. In this paper, a 6-DOF measurement method based on multi-camera is proposed, which is accomplished using at least two ordinary cameras and is made available for most morphological rigid bodies. First, multi-camera calibration based on Zhang Zhengyou’s calibration method is introduced. In addition to the intrinsic and extrinsic parameters of cameras, the pose relationship between the camera coordinate system and the world coordinate system can also be obtained. Secondly, the 6-DOF calculation model of proposed method is gradually analyzed by the matrix analysis method. With the help of control points arranged on the rigid body, the 6-DOF of the rigid body can be calculated by the least square method. Finally, the Phantom 3D high-speed photogrammetry system (P3HPS) with an accuracy of 0.1 mm/m was used to evaluate this method. The experiment results show that the average error of the rotational degrees of freedom (DOF) measurement is less than 1.1 deg, and the average error of the movement DOF measurement is less than 0.007 m. In conclusion, the accuracy of the proposed method meets the requirements.

## 1. Introduction

The measurement of six-degrees-of-freedom (6-DOF) is important in industrial production and the 6-DOF of a measured object represents its position information, which will help the machine to operate efficiently, thus 6-DOF measurements are often used in fields such as precision machining, spacecraft docking, and manufacturing assembly [1].

The 6-DOF of a rigid body include the rotational degrees of freedom (*Ψ*, *θ*, *φ*) around the *x*, *y*, and *z* axes as well as the movement degrees of freedom ($T_{x}$, $T_{y}$, $T_{z}$) along the *x*, *y*, and *z* axes. The commonly used measuring instruments or methods include laser, Hall sensor, inertial measurement unit (IMU), total station, and vision. Laser measurement methods, which include laser interferometer [2], laser tracker [3], and laser collimation method [4], have high accuracy, but a special optical path needs to be designed using a lens [5,6,7]. As a result, it has requirements on the size or range of motion of the measured object. In addition, the refractive index of the laser is susceptible to environmental factors such as humidity and temperature, which may cause errors [8,9]. Finally, specialized instruments such as laser trackers and laser interferometers are used for measurement in laser methods, which limits the widespread use of such methods [10,11]. In addition to the laser measurement, the Hall sensor is often used as a position sensing device for 6-DOF measurement [12] and to sense the 6-DOF in different positions and directions [13]. The accuracy of the Hall sensor is high, but most of them can only be used for micro measurement. On the other hand, when multiple Hall sensors are used for measurement, it is necessary to assemble the position of each sensor [14], so it is very necessary to calibrate the sensors, which is still a problem [15,16]. After assemble, the Hall sensor is fixed, which results in the inability to measure large objects or poor environmental adaptability [17,18,19]. Total stations are often used for measurement in long-distance and engineering environments, and have the characteristics of low cost and strong environmental adaptability. However, the total station alone cannot achieve dynamic 6-DOF measurement [5], and requires the use of overly complex cooperation goals [20]. In addition, there are measurement methods that use tools such as inertial measurement unit (IMU) [21] and laser scanning [22]. However, they are often used in combination with other sensors because of some limitations.

Compared with the above-mentioned 6-DOF measurement instruments or methods, the vision method has the advantages of non-contact, high accuracy, and wide measurement range [23,24,25,26]. With the development of image processing and deep learning, visual measurement methods have strong environmental adaptability [27,28]. Vision measurement can be divided into monocular [29,30,31] and multi-vision [32,33] measurement systems. The monocular vision measurement system has low hardware complexity and the shooting field of view is large [34,35], but it is difficult to measure the depth accurately [36]. For example, Hui Pan [37] proposed an estimation algorithm of relative pose of cooperative space targets based on monocular vision imaging, in which a modified gravity model approach and multiple targets tracking methods were used to improve the accuracy. In his experimental result, the translational error along the *z*-axis was obviously greater than that of the other two axes because of the module of monocular vision. On the other hand, monocular vision measurement of 6-DOF often converts into a Perspective-n-Point (PnP) problem, which requires knowing the coordinates of some points in the measured object coordinate system, and it only calculates the 6-DOF of the measured object in the camera coordinate system [38]. For example, Gangfeng Liu [39] proposed a monocular vision pose measurement method, which uses the guide petals of the docking mechanism to calculate the relative pose. In his work, it is necessary to extract the guide petals and obtain the pixel coordinates of key points to solve the PnP problem. If the measured object is changed, the proposed method will not be able to measure because it cannot extract the other measured objects.

Compared with monocular vision measurement, multi-vision measurement is more versatile. Multi-vision can measure depth more accurately, which is different from monocular vision [40]. Zhiyuan Niu [41] proposed an immersive positioning and measuring method based on multi-camera and designed the active light emitting diode (LED) markers as control points to deal with complicated industrial environment. However, the measurement result of this method is the pose between the measured object coordinate system and the camera coordinate system. The same situation also occurs in monocular vision measurement [39]. In this case, as long as the camera is moved, the 6-DOF of the measured object will be lost and the measurement needs to be performed again. In addition, the movement of the camera makes it difficult to reproduce the position and posture relationship between the measured object and the camera. On the other hand, some methods have a cooperative target that is complicated [40,41,42]. As a result, it has an influence on the versatility of the method. In multi-vision, the calibration between cameras is the key link, which determines the accuracy of measurements [43]. The commonly used calibration methods include the Zhang Zhengyou calibration method, Direct Linear Transformation (DLT) method, and so on, among which the Zhang Zhengyou calibration method is widely used because of its ease of operation and accuracy.

According to the above situation and problems, it is very necessary to propose a measurement method that is versatile, does not require professional instruments, and is suitable for most of the measured objects. Therefore, this paper proposes a multi-camera measurement method of 6-DOF for rigid bodies in the world coordinate system. First, multi-camera calibration based on Zhang Zhengyou’s calibration method is introduced. In addition to the intrinsic and extrinsic parameters of multiple cameras, it uses a checkerboard to calibrate the relationship between the camera coordinate system and the world coordinate system. Secondly, a universal measurement method of 6-DOF is proposed, and it only needs to arrange at least four non-coplanar control points on the rigid body. The coordinates of the control points in the rigid body coordinate system and the pixel coordinates on the image are used to calculate the 6-DOF of the rigid body. The 6-DOF measured by this method is the pose of the measured rigid body relative to the world coordinate system, which is not affected by the movement of cameras. Theoretically, the proposed method is suitable for dynamic and static measurement, but in order to better explain the principle and versatility of the method, only the static measurement is introduced.

The rest of this article is structured as follows. In Section 2, the principle of camera calibration and 6-DOF measurement is introduced. In Section 3, experiments are carried out and results are discussed. In Section 4, a summary is provided.

## 2. Principle and Methods

### 2.1. 6-DOF Basic Formula for Rigid Body

In space, an unrestricted rigid body has 6-DOF, which are rotation DOF around the *x*, *y*, and *z* axes as well as movement DOF along the three axes. The three DOF of movement are usually represented by the translation vector T, and the three DOF of rotation (three Euler angles) are represented by the rotation matrix R. Here, the rotation matrix R is a 3 × 3 order unit orthogonal matrix. In this paper, measuring the 6-DOF of a rigid body is performed to solve the rotation and movement DOF between the world coordinate system W (W system) and the rigid body coordinate system B (B system). According to the above, the rotation matrix $R_{B}^{W}$ and the translation vector $T_{B/W}^{W}$ between the two coordinate systems can be solved first, and then $R_{B}^{W}$ can be converted into three Euler angles. The form of $R_{B}^{W}$ is as follows:

(1) $$R_{B}^{W}=\left[\begin{array}{ccc} r_{11} & r_{12} & r_{13}\\ r_{21} & r_{22} & r_{23}\\ r_{31} & r_{32} & r_{33} \end{array}\right]\;,$$

The nine elements in the above matrix have the following relationship:

(2) $$\{\begin{array}{c} r_{11}r_{12}+r_{21}r_{22}+r_{31}r_{32}=0\\ r_{11}r_{13}+r_{21}r_{23}+r_{31}r_{33}=0\\ r_{12}r_{13}+r_{22}r_{23}+r_{32}r_{33}=0\\ r_{11}^{2}+r_{21}^{2}+r_{31}^{2}=1\\ r_{12}^{2}+r_{22}^{2}+r_{32}^{2}=1\\ r_{13}^{2}+r_{23}^{2}+r_{33}^{2}=1 \end{array}\;,$$

According to the *z*–*y*–*x* rotation order, it can be named in turn as the rotation angle *Ψ* around the *z*-axis, the rotation angle *θ* around the *y*-axis, and the rotation angle *φ* around the *x*-axis. The conversion relationship between the elements of $R_{B}^{W}$ and the three rotation angles (*Ψ*, *θ*, *φ*) is shown as follows:

(3) $$\{\begin{array}{c} \Psi =arctan\frac{r_{21}}{r_{11}}\\ \theta =arctan\left(-\frac{r_{31}}{\sqrt{r_{32}^{2}+r_{33}^{2}}}\right)\\ \varphi =arctan\frac{r_{32}}{r_{33}} \end{array}\;,$$

### 2.2. Pinhole Camera Model

**Figure 1:**
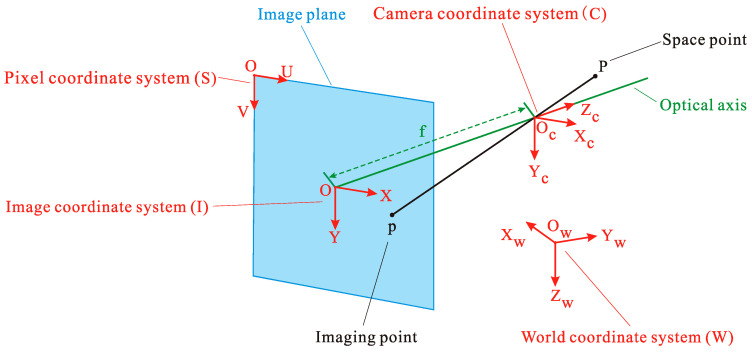
Pinhole camera model.

The imaging process of the camera can be regarded as the pinhole camera model. The three-dimensional (3D) scene is projected to the two-dimensional (2D) image plane. There is a certain mapping relationship during imaging, which can be simplified to perspective projection (Figure 1). The method proposed in this article and the explanation of subsequent principles are all based on the ideal pinhole camera model. Accordingly, the relationship between the pixel coordinates of the spatial point *P* in the image plane and its coordinates in the W system can be expressed as Equation (4) [44].

(4) $$z^{C}\left[\begin{array}{c} u\\ v\\ 1 \end{array}\right]=\left[\begin{array}{cccc} f_{x} & 0 & u_{0} & 0\\ 0 & f_{y} & v_{0} & 0\\ 0 & 0 & 1 & 0 \end{array}\right]\left[\begin{array}{cc} R_{W}^{C} & T_{W/C}^{C}\\ 0 & 1 \end{array}\right]\left[\begin{array}{c} x^{W}\\ y^{W}\\ z^{W}\\ 1 \end{array}\right]\;,$$

where ${\left(u\;,\;v\;,\;1\right)}^{T}$ is the homogeneous pixel coordinates of the imaging point *p*; ${\left(\begin{array}{c} x^{w}\;,\;y^{w}\;,\;z^{w}\;,\;1 \end{array}\right)}^{T}$ is the homogeneous world coordinates of the space point *P*; $z^{C}$ is the coordinate of the space point P in the *z*-axis direction of the camera coordinate system C (C system); $f_{x}=\frac{f}{d_{x}}$ and $f_{y}=f/d_{y}$, where $d_{x}$ and $d_{y}$ are the size of unit pixel on the *x*-axis and *y*-axis and f is the focal length; and $\left(u_{0}\;,v_{0}\right)$ is the pixel coordinate of the optical center, also known as the coordinate of the principal point. In the above, $f_{x}$, $f_{y}$, $u_{0}$, and $v_{0}$ refer to the intrinsic parameters of the camera, while $R_{W}^{C}$ and $T_{W/C}^{C}$ refer to the extrinsic parameters of the camera, which are the rotation matrix and translation vector from the W system to the C system. It shows a need to be explicit about exactly what is meant by the elements of $T_{W/C}^{C}$, which are the components of the vector in the W system.

### 2.3. Multi-Camera Calibration

Camera calibration is an indispensable step in vision measurement, which determines the accuracy of extracting 3D information of spatial points from 2D images. This method uses at least two cameras to measure 6-DOF, so it is necessary to estimate the intrinsic and extrinsic parameters of cameras. In addition, in order to simplify the subsequent calculation of the 6-DOF, this paper also calibrates the pose relationship between the W system and one of the C systems. On the basis of Zhang Zhengyou’s calibration method, this calibration called multi-camera calibration only uses a checkerboard pasted on a plate [45,46].

If the cameras are numbered according to any rules, the reference camera is described as the camera with number 1, and the non-reference cameras are described as the cameras with other numbers. Multi-camera calibration can best be treated under the following two parts: Part 1 is the calibration of the pose relationship between the non-reference cameras and the reference camera. This part of the calibration can obtain the intrinsic parameters of each camera, and the pose relationship between the reference camera coordinate system $C_{1}$ ($C_{1}$ system) and the non-reference camera coordinate system. Part 2 is the calibration of the pose relationship between the $C_{1}$ system and the W system. This part of the calibration can obtain the pose relationship ($R_{C_{1}}^{W}$, $T_{C_{1}/W}^{W}$) between the $C_{1}$ system and the W system.

**Figure 2:**
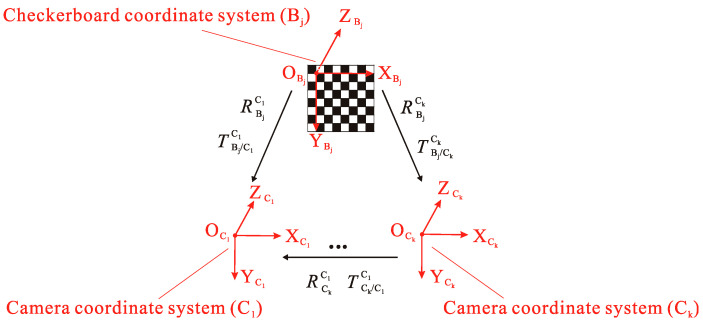
Multi-camera calibration.

In Part 1 of the calibration principle, in order to estimate the intrinsic parameters of each camera by Zhang Zhengyou’s calibration method, synchronous shooting by n (n ≥ 2) cameras obtains m (m ≥ 3) checkerboard images with different directions. Meanwhile, the pose relationship between the coordinate system $B_{j}$ (j = 1, 2 …, m) ($B_{j}$ system) established on the checkerboard and each camera coordinate system. Figure 2 shows a schematic diagram of using a checkerboard to calibrate. While P is an arbitrary corner point on the checkerboard, its coordinate in the $B_{j}$ system is described as $P^{B_{j}}$. In the $C_{1}$ system and $C_{k}$(*k* = 2, 3..., n) system, the coordinates of *P* are described as $P^{C_{1}}$ and $P^{C_{k}}$. The conversion relationship between $P^{B_{j}}$, $P^{C_{1}}$, $P^{C_{k}}$ can be expressed as follows:

(5) $$P^{C_{1}}=R_{B_{j}}^{C_{1}}P^{B_{j}}+T_{B_{j}/C_{1}}^{C_{1}}\;,\;P^{C_{k}}=R_{B_{j}}^{C_{k}}P^{B_{j}}+T_{B_{j}/C_{k}}^{C_{k}}\;,$$

where $R_{B_{j}}^{C_{1}}$ and $R_{B_{j}}^{C_{k}}$ represent the rotation matrix from the $B_{j}$ system to the $C_{1}$ and $C_{k}$ systems; and $T_{B_{j}/C_{1}}^{C_{1}}$ and $T_{B_{j}/C_{k}}^{C_{k}}$ represent the translation vector from the $B_{j}$ system to the $C_{1}$ and $C_{k}$ systems, and its elements are the components of the vector in the $C_{1}$ and $C_{k}$ systems.

Eliminating $P^{B_{j}}$ in Equation (5), the following indicates the conversion relationship between $P^{C_{1}}$ and $P^{C_{k}}$:

(6) $$P^{C_{1}}=R_{B_{j}}^{C_{1}}{\left(R_{B_{j}}^{C_{k}}\right)}^{-1}P^{C_{k}}+T_{B_{j}/C_{1}}^{C_{1}}-R_{B_{j}}^{C_{1}}{\left(R_{B_{j}}^{C_{k}}\right)}^{-1}T_{B_{j}/C_{k}}^{C_{k}},$$

Assuming $\left(\begin{array}{cc} R_{C_{k}}^{C_{1}} & T_{C_{k}/C_{1}}^{C_{1}} \end{array}\right)=\left(\begin{array}{cc} R_{B_{j}}^{C_{1}}{\left(R_{B_{j}}^{C_{k}}\right)}^{-1} & T_{B_{j}/C_{1}}^{C_{1}}-R_{B_{j}}^{C_{1}}{\left(R_{B_{j}}^{C_{k}}\right)}^{-1}T_{B_{j}/C_{k}}^{C_{k}} \end{array}\right)$, the above equation can be abbreviated as follows:

(7) $$X_{1}=R_{C_{k}}^{C_{1}}X_{k}+T_{C_{k}/C_{1}}^{C_{1}}\;,$$

where $\left(\begin{array}{cc} R_{C_{k}}^{C_{1}} & T_{C_{k}/C_{1}}^{C_{1}} \end{array}\right)$ is the rotation matrix and translation vector from the $C_{k}$ system to the $C_{1}$ system.

**Figure 3:**
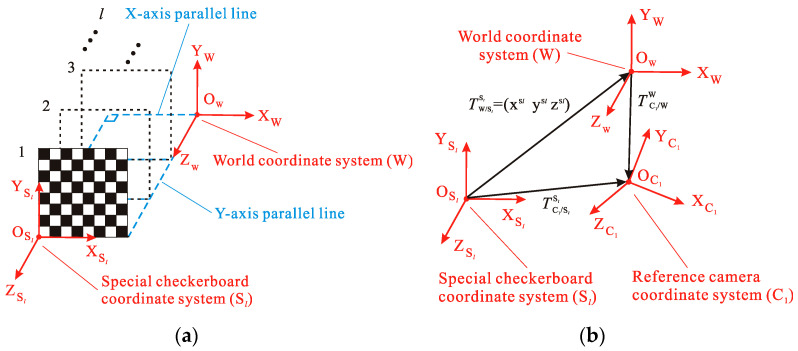
The location and principle of the special checkerboard: (**a**) relationship of the rotation matrix; (**b**) relationship of the translation vector.

In Part 2 of the calibration principle, there are m different $B_{j}$ systems in the m checkerboard images with different orientations, among which there are l (3≤l≤m) checkerboard coordinate systems $B_{j}$, which have the following relationship with the W system: (1) the coordinate axis direction of the $B_{j}$ system is consistent with the W system in its corresponding position; (2) the coordinates of the origin of the W system in the $B_{j}$ system are known. The $B_{j}$ system that conforms to the above-mentioned relationship is called the special checkerboard coordinate system $S_{l}$ ($S_{l}$ system), and the relationship is shown in Figure 3a. As mentioned above, it can be seen that the pose relationship between the $S_{l}$ system and the W system is as follows:

(8) $$\{\begin{array}{c} R_{S_{l}}^{W}=I\\ T_{W/S_{l}}^{S_{l}}=\left(x^{S_{l}},\;\;y^{S_{l}},\;\;z^{S_{l}}\right) \end{array}\;,$$

where $R_{S_{l}}^{W}$ is the rotation matrix from the $S_{l}$ system to the W system; ***I*** is the 3 × 3 identity matrix; $T_{W/S_{l}}^{S_{l}}$ is the translation vector from the W system to the $S_{l}$ system, and its elements are the components of the vector in the $S_{l}$ system; and is the origin of the W system, which is represented by the coordinate of the $S_{l}$ system.

Figure 3b shows the geometric relationship of the translation vectors among some coordinate systems, and the geometric relationship can be expressed as follows:

(9) $$T_{C_{1}/W}^{W}=T_{C_{1}/S_{l}}^{S_{l}}-T_{W/S_{l\;}}^{S_{l}},$$

In the calibration process of Part 1, $R_{S_{l}}^{C_{1}}$ and $T_{S_{l}/C_{1}}^{C_{1}}$ corresponding to the $S_{l}$ system and the $C_{1}$ system are known. In order to obtain the pose relationship between the $C_{1}$ system and the W system, Equation (10) can be obtained by combining Equations (8) and (9). So far, the multi-camera calibration is completed. The intrinsic parameters of each camera, $R_{C_{1}}^{W}$ and $T_{C_{1}/W}^{W}$ between the $C_{1}$ system and the W system are obtained. According to the above principle, $R_{C_{1}}^{W}$ is calculated, but it is unknown whether $R_{C_{1}}^{W}$ is an orthogonal matrix. Therefore, the following works must to be performed, which are based on singular value decomposition (SVD) [47]: (1) assuming $\hat{R_{C_{1}}^{W}}$ is the $R_{C_{1}}^{W}$ calculated above, calculating by SVD, we can obtain $\hat{R_{C_{1}}^{W}}=UDV^{T}$; (2) D is a diagonal matrix and its elements are singular values of $\hat{R_{C_{1}}^{W}}$, the singular values of a 3 × 3 orthogonal matrix are all 1; (3) changing D by an identity matrix ***I***, we can obtain $R_{C_{1}}^{W}=UIV^{T}$, which is orthogonal.

(10) $$\{\begin{array}{c} R_{C_{1}}^{W}=R_{C_{1}}^{S_{l}}R_{S_{l}}^{W}={\left(R_{S_{l}}^{C_{1}}\right)}^{-1}={\left(R_{S_{l}}^{C_{1}}\right)}^{T}\\ T_{C_{1}/W}^{W}=T_{C_{1}/S_{l}}^{S_{l}}-T_{W/S_{l}}^{S_{l}} \end{array}.$$

### 2.4. 6-DOF Measurement of Rigid Body in World Coordinate System

It can be seen from Section 2.1 that measuring the 6-DOF of a rigid body is performed to calculate the rotation matrix $R_{B}^{W}$ and the translation vector $T_{B/W}^{W}$ between the W system and the B system. Suppose there are *i* control points $P_{i}$ on the measured rigid body, among which there are at least four non-coplanar control points. To ensure the accuracy of the measurement, the control points should be evenly distributed on the rigid body, covering the entire main structure of the rigid body. On the basis of the above-mentioned, $R_{B}^{W}$ and $T_{B/W}^{W}$ can be obtained from Equation (11).

(11) $$P_{i}^{W}=R_{B}^{W}P_{i}^{B}+T_{B/W}^{W}\;,$$

The above equation is converted into homogeneous coordinate form as follows:

(12) $$P_{i}^{W}=\left[\begin{array}{cc} R_{B}^{W} & T_{B/W}^{W} \end{array}\right]\left[\begin{array}{c} P_{i}^{B}\\ I \end{array}\right]\;,$$

In Equation (12), If $P_{i}^{B}$ and $P_{i}^{W}$ are known, $R_{B}^{W}$ and $T_{B/W}^{W}$ can be calculated. As $P_{i}$ is on the measured rigid body, its coordinates $P_{i}^{B}$ in the B system can be obtained by manual measurement. The coordinates $P_{i}^{W}$, which is the coordinates of $P_{i}$ in the W system, may difficult to measure by manual measurement because the W system is set independently. Fortunately, $P_{i}^{W}$. can be measured according to the following principle.

**Figure 4:**
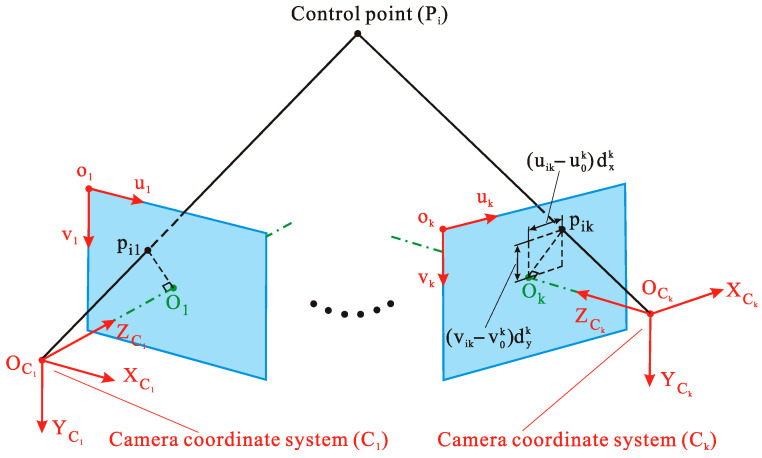
The coordinate solution of $P_{i}$ in the reference camera coordinate system $C_{1}$.

According to the assumptions in Section 2.3, the reference camera and the non-reference cameras are defined. This section is based on the above assumption. In the measurement of $P_{i}^{W}$, the pixel coordinates of the imaging point $p_{ik}$ of $P_{i}$ on the camera k are applied to the calculation of the $P_{i}^{C_{1}}$ represented by the coordinate of $C_{1}$ system of $P_{i}$. Following this, $R_{C_{1}}^{W}$ and $T_{C_{1}/W}^{W}$ obtained in Section 2.3 are used to calculate $P_{i}^{W}$ of $P_{i}$ in the W system. The schematic diagram of the coordinate solution of $P_{i}$ in the $C_{1}$ system is shown in Figure 4. $p_{ik}$ is the imaging point of the control point $P_{i}$ on the image plane of camera k, and $\left(u_{ik},v_{ik}\right)$ is the pixel coordinate of $p_{ik}$. In camera k, the pixel coordinate of the intersection (principal point) $O_{k}$ of the optical axis and the image plane is defined as $\left(u_{0}^{k},v_{0}^{k}\right)$, which is the principal point coordinates of camera k. According to Figure 4, there is the relationship below of the coordinate conversion of $P_{i}$ from the $C_{1}$ system to the $C_{k}$ system.

(13) $$P_{i}^{C_{1}}=R_{C_{k}}^{C_{1}}P_{i}^{C_{k}}+T_{C_{k}/C_{1}}^{C_{1}}\;,$$

As shown in Figure 4, theoretically, in camera k, points $P_{i}$, $p_{ik}$, and $O_{C_{k}}$ are on the same straight line, and $\vec{O_{C_{k}}P_{i}}=s_{k}\vec{O_{C_{k}}p_{ik}}$; thereby, Equation (13) can be transformed into Equation (14). In addition, according to the geometric relationship between the imaging point $p_{ik}$ and the principal point, $p_{ik}$ of the *i*-th control point $P_{i}$ has the 3D coordinates $p_{ik}^{C_{k}}={\left[\;\left(u_{ik}-u_{0}^{k}\right)d_{x}^{k}\;,\;\left(v_{ik}-v_{0}^{k}\right)d_{y}^{k}\;,\;f_{k}\;\right]}^{T}$, where $d_{x}^{k}$ and $d_{y}^{k}$ are the sizes of unit pixel of camera *k* in the X and Y axis, and $f_{k}$ is the focal length of camera *k*.

(14) $$P_{i}^{C_{1}}=S_{k}R_{C_{k}}^{C_{1}}p_{ik}^{C_{k}}+T_{C_{k}/C_{1}}^{C_{1}}\;,$$

where $P_{i}^{C_{1}}$ and $P_{i}^{C_{k}}$ are the 3D coordinates of $P_{i}$ in the $C_{1}$ system and the $C_{k}$ system; $R_{C_{k}}^{C_{1}}$ and $T_{C_{k}/C_{1}}^{C_{1}}$, calculated by Equation (7) in Section 2.3, are the rotation matrix and translation vector from the $C_{k}$ system to the $C_{1}$ system; $S_{k}$ is the scale factor; and $p_{ik}^{C_{k}}$ is the 3D coordinate of the imaging point $p_{ik}$ of the i-th control point $P_{i}$ in the $C_{k}$ system.

In Equation (14), $P_{i}^{C_{1}}$ and $S_{k}$ are unknown quantities. For a single camera, while three equations can be listed, there are four unknowns; thereby, the unknown quantities cannot be measured. For *k*(*k* ≥ 2) cameras, there are (*k* + 3) unknowns quantities, and the number of equations is 3*k*. In this case, $P_{i}^{C_{1}}$ and $S_{k}$ can be calculated by the least square method. Equation (14) is now converted into matrix form because of the convenient calculation:

(15) $$\left[\begin{array}{cc} I & -R_{C_{k}}^{C_{1}}p_{ik}^{C_{k}} \end{array}\right]\left[\begin{array}{c} P_{i}^{C_{1}}\\ S_{k} \end{array}\right]=T_{C_{k}/C_{1}}^{C_{1}}\;,$$

For *k* cameras, 3*k* linear equations can be listed and expressed as equations:

(16) Ax=b ,

In Equation (16),

(17) $$\begin{array}{c} A=\left[\begin{array}{ccccc} I & -p_{i1}^{C_{1}} & 0 & \ldots & 0\\ I & 0 & -R_{C_{2}}^{C_{1}}p_{i2}^{C_{2}} & \ldots & 0\\ \vdots & \vdots & \vdots & \vdots & \vdots \\ I & 0 & 0 & \ldots & -R_{C_{k}}^{C_{1}}p_{ik}^{C_{k}} \end{array}\right]\\ \begin{array}{c} x={\left[\begin{array}{ccccc} P_{i}^{C_{1}} & S_{1} & S_{2} & \cdots & S_{k} \end{array}\right]}^{T}\;,\\ b={\left[\begin{array}{cccc} 0_{3\times 1} & T_{C_{2}/C_{1}}^{C_{1}} & \cdots & T_{C_{k}/C_{1}}^{C_{1}} \end{array}\right]}^{T}\;, \end{array} \end{array}\;\;,$$

The least square method can be used to solve for x in the following equation:

(18) $$x={\left(A^{T}A\right)}^{-1}A^{T}b\;,$$

Here, the 3D coordinate $P_{i}^{C_{1}}$ of the control point $P_{i}$ in the $C_{1}$ system is calculated, and then the pose relationship $R_{C_{1}}^{W}$, $T_{C_{1}/W}^{W}$ from the $C_{1}$ system to the W system were calculated in Equation (10), and they are used in Equation (19) to convert the 3D coordinate $P_{i}^{C_{1}}$ into the 3D coordinate $P_{i}^{W}$.

(19) $$P_{i}^{W}=R_{C_{1}}^{W}P_{i}^{C_{1}}+T_{C_{1}/W}^{W}\;,$$

Isummary, the 3D coordinate $P_{i}^{W}$ of the control point $P_{i}$ in the W system was calculated by the above principle, and the 3D coordinate $P_{i}^{B}$ of the control point $P_{i}$ in the B system was obtained by manual measurement. $P_{i}^{B}$ are denoted by $P_{i}^{B}=\left(x_{i}^{B}\;,\;y_{i}^{B}\;,\;z_{i}^{B}\right)$, and $P_{i}^{W}$ are denoted by $P_{i}^{W}=\left(x_{i}^{W}\;,\;y_{i}^{W}\;,\;z_{i}^{W}\right)$; Equation (11) then illustrates the coordinate relationship of $P_{i}$ in the above two coordinate systems. If $x={\left(\begin{array}{cccccccc} r_{11} & r_{12} & r_{13} & \cdots & r_{33} & T_{x} & T_{y} & T_{z} \end{array}\right)}^{T}$, where r is the components of $R_{B}^{W}$ and $\left(\begin{array}{ccc} T_{x} & T_{y} & T_{z} \end{array}\right)$ is the components of $T_{B/W}^{W}$, then for each $P_{i}$ (the number of non-coplanar control points is at least four), all have the following:

(20) $$\left[\begin{array}{c} x_{i}^{W}\\ y_{i}^{W}\\ z_{i}^{W} \end{array}\right]=\left[\begin{array}{cccccccccccc} x_{i}^{B} & y_{i}^{B} & z_{i}^{B} & 0 & 0 & 0 & 0 & 0 & 0 & 1 & 0 & 0\\ 0 & 0 & 0 & x_{i}^{B} & y_{i}^{B} & z_{i}^{B} & 0 & 0 & 0 & 0 & 1 & 0\\ 0 & 0 & 0 & 0 & 0 & 0 & x_{i}^{B} & y_{i}^{B} & z_{i}^{B} & 0 & 0 & 1 \end{array}\right]x\;,$$

For i control points, the least square method can be used to calculate x:

(21) $$\begin{array}{c} Ax=b\\ x={\left(A^{T}A\right)}^{-1}A^{T}b\; \end{array},$$

Here,

(22) $$\begin{array}{c} A=\left[\begin{array}{cccccccccccc} x_{1}^{B} & y_{1}^{B} & z_{1}^{B} & 0 & 0 & 0 & 0 & 0 & 0 & 1 & 0 & 0\\ 0 & 0 & 0 & x_{1}^{B} & y_{1}^{B} & z_{1}^{B} & 0 & 0 & 0 & 0 & 1 & 0\\ 0 & 0 & 0 & 0 & 0 & 0 & x_{1}^{B} & y_{1}^{B} & z_{1}^{B} & 0 & 0 & 1\\ \vdots & \vdots & \vdots & \vdots & \vdots & \vdots & \vdots & \vdots & \vdots & \vdots & \vdots & \vdots \\ x_{i}^{B} & y_{i}^{B} & z_{i}^{B} & 0 & 0 & 0 & 0 & 0 & 0 & 1 & 0 & 0\\ 0 & 0 & 0 & x_{i}^{B} & y_{i}^{B} & z_{i}^{B} & 0 & 0 & 0 & 0 & 1 & 0\\ 0 & 0 & 0 & 0 & 0 & 0 & x_{i}^{B} & y_{i}^{B} & z_{i}^{B} & 0 & 0 & 1 \end{array}\right]\;,\\ b={\left[\begin{array}{ccccccc} x_{1}^{W} & y_{1}^{W} & z_{1}^{W} & \cdots & x_{i}^{W} & y_{i}^{W} & z_{i}^{W} \end{array}\right]}^{T}\;, \end{array}$$

Here, the pose relationship parameters $R_{B}^{W}$. and $T_{B/W}^{W}$ between the B system and the W system were calculated. To ensure the orthogonality of the rotation matrix $R_{B}^{W}$, the obtained $\hat{R_{B}^{W}}=UDV^{T}$ needs to be calculated by singular value decomposition (SVD), and ***D*** is replaced by unit matrix ***I***. Now, $R_{B}^{W}=UIV^{T}$, so as to ensure the orthogonality of the rotation matrix. After that, $R_{B}^{W}$ is converted into three rotational DOF according to Section 2.1. So far, the degree of freedom of movement and rotation of the rigid body have been calculated.

### 2.5. 6-DOF Measurement Method

**Figure 5:**
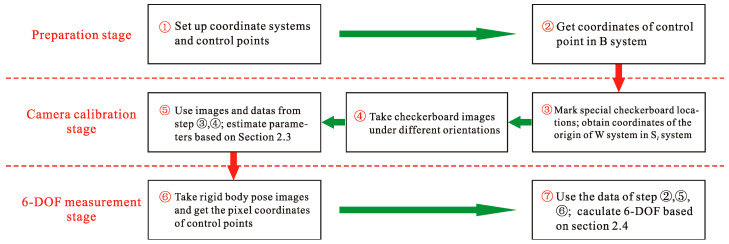
A universal method for six-degrees-of-freedom (6-DOF) measurement of rigid bodies.

According to the principle described in Chapter 2, a universal method for measuring 6-DOF of rigid bodies in the world coordinate system based on multiple cameras can be summarized, including three stages: preparation, camera calibration, and 6-DOF measurement. The proposed method including seven steps is shown in Figure 5.

#### 2.5.1. Preparation Stage

Step 1. Establish the W system and B system in place as required. The origin of the B system is usually set at the center of mass of the measured rigid body. The B system is fixed to the rigid body and moves with the movement of the rigid body. In addition, the aforementioned W system and B system are both right-handed coordinate systems. The *i* control points $P_{i}$ are evenly distributed on the measured rigid body, among which at least four control points are not coplanar.

Step 2. Use a scale or other simple tools to measure the 3D coordinate $P_{i}^{B}$ (rigid body coordinate) of $P_{i}$. in the B system. The 3D coordinate $P_{i}^{B}$ can be measured multiple times and the average value can be calculated to reduce errors. The coordinate data measured in this step will be used in step 7 to calculate the 6-DOF. Next, it is necessary to set up and connect the camera in a proper position. After adjusting, the camera number can be specified. The camera numbered 1 is the reference camera, and others are non-reference cameras.

#### 2.5.2. Camera Calibration Stage

Step 3. Mark the position of the special checkerboard, and place the checkerboard at the position marked in advance during calibration. Here, the special checkerboard $S_{l}$ system and W system established on the checkerboard conform to the relationship of Equation (8). In order to mark the position of the special checkerboard, some tools such as line segment laser measuring instrument and the guide rail can be used to draw the line on the ground. Once the marking is completed, the coordinates of the origin of the W system in the $S_{l}$ system are measured by scale or other tools.

Step 4. Multiple cameras synchronously take a number of checkerboard images with different orientations (at least 10 images for each camera). Step by step, place the checkerboard in the marked positions of the special checkerboard. The next step will use the special checkerboard images to calibrate the pose relationship between the $C_{1}$. system and the W system. Following this, the experimenter holds the checkerboard and moves slowly in the shooting space when multiple cameras continue to synchronously capture checkerboard images. During this period, the checkerboard plane always faces multiple cameras so that the checkerboard is as full as possible in the public field of view of the camera.

Step 5. Select the eligible images from the checkerboard images taken in step 4, write the calibration code based on the principle of multi-camera calibration in Section 2.3 on the relevant software (MATLAB, OpenCV, and so on), and estimate the calibration parameters. Without considering the camera distortion, the calibration parameters include the intrinsic parameters of each camera ($f_{x}$, $f_{y}$, $u_{0}$, $v_{0}$), the pose relationship between the non-reference cameras coordinate system and the $C_{1}$. system (rotation matrices, translation vectors), and the pose relationship between the $C_{1}$. system and the W system (rotation matrix, translation vector). The calibration parameters will be used in step 7 to calculate 6-DOF.

#### 2.5.3. 6-DOF Measurement Stage

Step 6. After calibration, the camera cannot be moved, in order to ensure the whole rigid body is in the public field of view when multiple cameras synchronously shoot static images of rigid body (static measurement) or continuous motion images of rigid body (dynamic measurement). After the measurement images are taken, the pixel coordinates of the control points on the images taken by each camera are obtained. The pixel coordinates will be used in step 7 to calculate the 6-DOF.

Step 7. Use the data in step 2, 5, and 6, and write code to calculate the pose relationship between the W system and the B system (6-DOF of rigid body) based on the principle of Section 2.4. First, on the basis of the data in step 6 and Equations (15)–(18), the 3D coordinates $P_{i}^{C_{1}}$ of $P_{i}$ in the $C_{1}$ system are calculated using the least square method. Next, on the basis of the calibration parameters of step 5 ($R_{C_{1}}^{W}$ and $T_{C_{1}/W}^{W}$) and Equation (19), the 3D coordinates $P_{i}^{W}$ of $P_{i}$ in the W system are calculated. Then, on the basis of the coordinates $P_{i}^{B}$, Coordinates $P_{i}^{W}$ and Equation (11), $R_{B}^{W}$, $T_{B/W}^{W}$ between the W system and the B system are figured out using the least square method. Finally, on the basis of $R_{B}^{W}$, the three rotation DOF are calculated by Equation (3) in Section 2.1.

## 3. Experimental Results and Discussions

In order to evaluate the performance of the proposed method, this paper takes two cameras as an example and takes the shovel of a paddy field grader as the measured rigid body. The proposed method and the Phantom 3D high-speed photogrammetry system were used to measure the 6-DOF of the rigid body at the same time. As the measurement accuracy of Phantom 3D high-speed photogrammetry system (P3HPS) can reach 0.1 mm/m, the results of P3HPS were defined as the true values and the results of the proposed method were defined as the measured values. The absolute error was calculated by the equation $\mathrm{error}=\left|x-x_{0}\right|$, where x represented the measured values, and $x_{0}$ represented the true values. The experiment can best be treated under two parts: evaluate the performance of the proposed method by the P3HPS and the sensitivity of the proposed method at different measurement distances. In the first part, a static measurement experiment was carried out to verify the feasibility and accuracy of the proposed method. In the second part, the experiment on the influence of measuring distance on measuring accuracy was carried out, which provides a research basis for further improving the accuracy of the method.

### 3.1. Experimental Setup

P3HPS, which was produced by Vision Research company, includes a Phantom VEO 410 high-speed camera, a Phantom M310 high-speed camera, a one-dimensional calibration rod, lights, a laptop, camera control software PCC 3.1, 3D measurement software TEMA 4.0, and wiring harnesses. Some parameters of two high-speed cameras are shown in Table 1. Otherwise, the two high-speed cameras have the same lens, which is Nikon 24–85 mm f/2.8-4D AF Zoom. The focal length range of this lens is 24–85 mm, and the f-number range is F22–F2.8. In addition, the images obtained by the two high-speed cameras during the measurement was also used for the measurement of the proposed method. 

**Figure 6:**
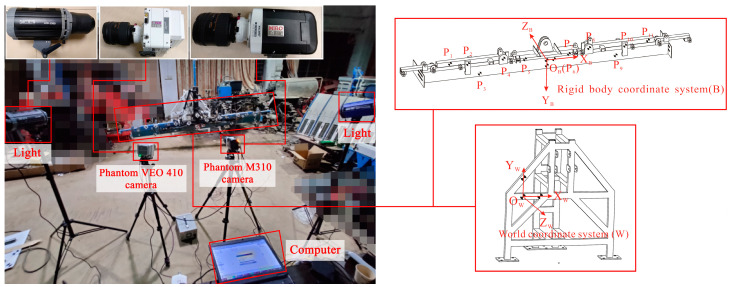
Experiment layout.

The shovel of a paddy field grader was defined as the measured rigid body and the size of the region of interest on the shovel is 3009 × 203 mm. The elevation and horizontal cylinders on the shovel were used to control the 6-DOF change of the rigid body. Eleven control points were arranged on the shovel to assist in measuring 6-DOF. To ensure the accuracy and stability of the measurement, the control points should be evenly distributed on the rigid body and cover the main structure of the rigid body. It needs to be emphasized that the arrangement of the control points was completed by pasting paper markers (similar to the BMW logo), mainly for the convenient use of the P3HPS to identify the control points. The layout of the experiment is shown in Figure 6. The printed checkerboard pattern (11 × 8 in size, 40 mm × 40 mm for a single small square) was pasted on the plate for calibration. When marking the position of a special checkerboard, a line segment laser measuring instrument was applied to assist in drawing lines on the ground, including parallel lines of *x* and *z* axes of the W system. Install PCC 3.1 on the computer and connect it with the camera by the wire harness, then turn on the PCC 3.1 on the computer to control the cameras to shoot simultaneously.

### 3.2. 6-DOF Measurement Analysis with P3HPS

#### 3.2.1. Camera Calibration

**Figure 7:**
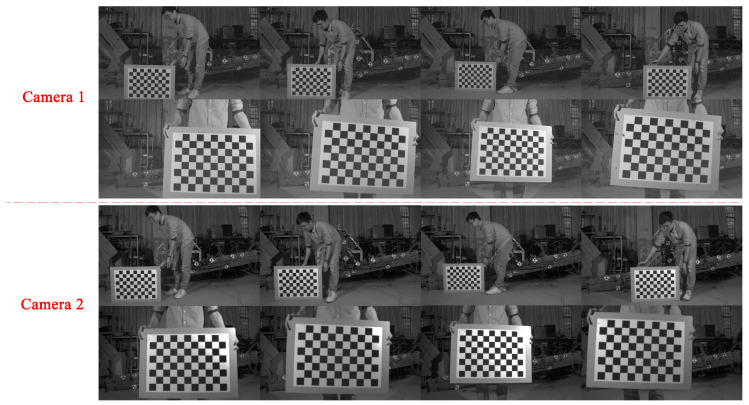
Part of the checkerboard images.

In the experiment environment of 6 m × 6 m × 2 m, two cameras were arranged at a distance of 3 m from the measured rigid body. The experiment operator gradually placed the checkerboard on the marked special checkerboard position and then held the checkerboard to rotate or move the checkerboard in space. Meanwhile, the two cameras simultaneously acquired images of the checkerboard. The experiment operator needed to move within the depth of field of the cameras to ensure that the checkerboard images taken are clear. The checkerboards in this experiment were all shot within 1–3 m from the cameras, which is within the depth of field. It is necessary to place the checkerboard at an angle less than 45° relative to the camera plane. In the experiment, a total of 20 pairs of suitable checkerboard images were taken for calibration, of which 7 pairs were special checkerboard images and the rest were non-special checkerboard images. Figure 7 shows a part of checkerboard images obtained during the experiment. The resolution of the checkerboard images was 1280 × 800, the f-number of the two cameras was f/22, and the focal length of the two cameras was 24 mm.

According to the principle of multi-camera calibration in Section 2.3 and the universal method in Section 2.5, the camera’s intrinsic parameters $\left(f_{x},\;f_{y},\;u_{0},\;v_{0}\right)$, the pose relationship between the cameras, and the pose relationship from the $C_{1}$ system to W system were estimated after multi-camera calibration. Different from other camera calibration, the pose relationship calibration between the W system and the $C_{1}$ system was carried out. Theoretically, the three rotational DOF from the $C_{1}$ system to the $S_{l}$ system in each special checkerboard image are the same. This condition can be used as a basis for judging whether a special checkerboard image is qualified. The intrinsic parameters of two cameras estimated by multi-camera calibration are shown in Table 2. The theoretical principal point coordinates of two cameras are (640, 400), but the principal point is offset as a result of errors in lens assembly, so the principal point coordinates in Table 2 are reasonable. The focal length we actually use is 24 mm and the focal length in the table is close to the real values, which preliminarily proves the reliability of the calibration results.

The intermediate quantities in the calibration process of the pose relationship between the W system and the $C_{1}$ system are set out in Table 3 and Table 4, where Table 3 shows the relevant data of the rotation matrix between the two coordinate systems, and Table 4 shows the relevant data of the translation vector between the two coordinate systems. The average values of rotational DOF around the *x*, *y*, and *z* axes between the W system and the $C_{1}$. system were −179.17°, −4.39°, and −9.90°, respectively, and the standard deviations around the three axes were 0.2121°, 0.1183°, and 0.3417° (Table 3), respectively, which was roughly consistent with the actual experiment scene. The low dispersion degree of the data also indicated that the calibration data are highly reliable, with the lowest dispersion degree in the *y*-axis direction, the second in the *x*-axis direction, and the highest dispersion degree in the *z*-axis direction. It can be seen from Table 4 that the average values of movement DOF along the *x*, *y*, and *z* axes between the W system and the $C_{1}$. system were −0.2663 m, 0.0712 m, and 3.6836 m, respectively, and the standard deviations were 0.0110 m, 0.0036 m, and 0.0145 m, respectively. The same as Table 3, the dispersion degree of the *z*-axis is the highest.

The results of the multi-camera calibration and the calibration results of P3HPS are compared in Table 5. The first two rows of Table 5 are the comparison of the calibration results $R_{C_{2}}^{C_{1}}$, $T_{C_{2}/C_{1}}^{C_{1}}$. The latter two rows are the comparison of the calibration results $R_{C_{1}}^{W}$, $T_{C_{1}/W}^{W}$. Here, the calibration results of the P3HPS measurement system were defined as the true values. On the one hand, regarding the calibration of the pose relationship between two cameras, the average errors of rotation vector and translation vector were 0.26° and 0.0028 m, respectively. As can be seen from the table below, the errors of the rotation vector and translation vector in the *z*-axis direction were the largest, while those in the *x*-axis direction were the smallest. Although the absolute error of the rotation vector and translation vector between the two cameras seems to be small, the relative error is significantly large. Therefore, we read the relevant work and check the equipment used in the experiment to analyze the reasons. In conclusion, it is caused by the following reasons: (1) The printed checkerboard has poor clarity, resulting in blurry corner points; the plane where the checkerboard was pasted is not an absolute plane, which leads to errors in corner extraction; and the size of a single small square in the checkerboard is inconsistent. (2) In the calibration images, the checkerboard has a large amount of movement in the depth direction. When the checkerboard is far from the camera, the checkerboard in the calibration image is too small, which makes the calibration inaccurate. (3) Because this paper is based on the pinhole imaging model, the multi-camera calibration does not consider the distortion, which makes the relative error large. One the other hand, regarding the calibration of the pose relationship between the W system and the $C_{1}$ system, the average errors of the rotation vector and translation vector were 0.32° and 0.0042 m. The error of rotation vector in the *z*-axis direction was the largest, while that in the *x*-axis direction was the smallest. The maximum and minimum errors of translation vector were opposite to the rotation vector. The accuracy of the calibration method can be considered to meet the requirements according to the maximum error and the average errors. On the basis of the above, the multi-camera calibration is practical and feasible, and the calibration of the pose relationship between the W system and the $C_{1}$ system is completed while calibrating the pose relationship between the cameras.

#### 3.2.2. Experimental Results for 6-DOF Measurement

On the basis of the calibration in Section 3.2.1, 50 static rigid body images with different poses were taken for 50 static measurements. Here, the positions of two camera were the same as that in Section 3.2.1. Otherwise, the accuracy of the proposed method was verified by P3HPS. The resolution of the captured images was 1280 × 800 and the cameras parameters settings were the same as in Section 3.2.1. The results of 50 measurements by the proposed method were calculated based on Section 2.4.

**Figure 8:**
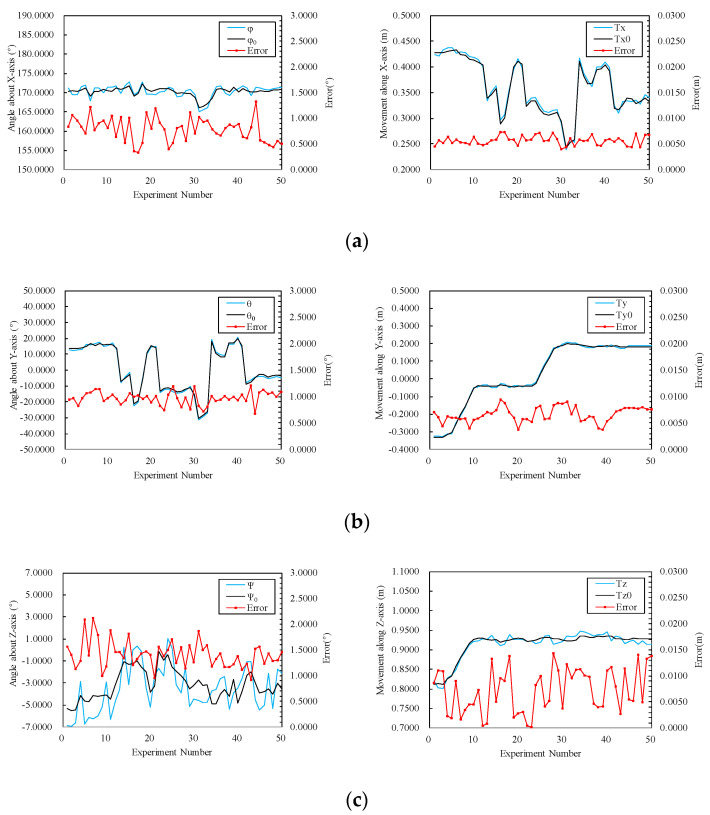
Measurement results and errors of 6-DOF (measurement distance is 3 m): (**a**) measurement results and errors in the *x*-axis direction; (**b**) measurement results and errors in the *y*-axis direction; (**c**) measurement results and errors in the *z*-axis direction.

In 50 static measurements, the average error of the rotational DOF measured by the proposed method was 1.0557° and the average error of the movement DOF was 0.0065 m. The standard deviations of the DOF of rotation and the DOF of movement were 0.3396° and 0.0027 m, respectively. If $\left(\varphi ,\;\theta ,\;\Psi ,\;T_{x},\;T_{y},\;T_{z}\right)$ was used to represent the measured values of the rotational DOF and movement DOF of the rigid body in *x*, *y*, and *z* axes, $\left(\varphi_{0},\;\theta_{0},\;\Psi_{0},\;T_{x0},\;T_{y0},\;T_{z0}\right)$ represented the true values of the 6-DOF of the rigid body. The average errors of each DOF and the standard deviations of errors can be calculated as set out in Table 6. By longitudinal comparison of the table, it can be found that the average error of the *z*-axis was the largest when measuring the rotational DOF, which can reach 1.4117°. The average error of the *z*-axis was also the largest when measuring the movement DOF, which can reach 0.0072 m. In addition, the standard deviations of the measurement errors of the rotational DOF and movement DOF in the *z*-axis direction were larger than those of the other two axes. Draw the true value, measured value, and the errors of 50 static measurements in the form of line diagrams, as shown in Figure 8. In 50 static measurements, the maximum errors of $\varphi ,\;\theta ,\;\Psi ,\;T_{x},\;T_{y},\;\mathrm{and}\;T_{z}$ were 1.3325°, 1.2058°, 2.1257°, 0.0073 m, 0.0094 m, and 0.0144 m, respectively, and the minimum errors were 0.3371°, 0.6730°, 0.9147°, 0.0040 m, 0.0037 m, and 0.0002 m, respectively. The longitudinal comparison results in Table 6 can be seen again by observing the degree of fit (Figure 8) between the measured values line (black) and the true values line (blue).

Combining Table 6 and Figure 8, the following two conclusions can be drawn: (1) the accuracy of the proposed method to measure the 6-DOF of rigid bodies is routine. In general, at a measuring distance of 3 m, the average error of the rotational DOF was better than 1.1°, and the average error of the movement DOF was better than 0.007 m. (2) The measurement errors and standard deviations of the movement DOF and rotational DOF in the *z*-axis direction were larger than those in the other two directions. From this point of view, the measurement accuracy and stability in the *z*-axis direction are slightly worse than the other two directions, which is the same as in [41]. In measurement, the camera’s depth of field was constant. Because the range of depth of field is limited, the control points on the rigid body may not be able to shoot clearly as the measured rigid body leaves the range of depth of field, which results in errors. Furthermore, the measured values in the *z*-axis direction are the largest. When we use Equation (14) to calculate, the error amplification in the *z*-axis direction will be larger than in the other two directions. In addition, the f-number, focal length, and distance of the focused object will all have an effect on the depth of field. A smaller f-number and shorter focal length will have a larger depth of field. In the experiment, we have used the smallest f-number and focal length. If a lens with a smaller f-number and focal length is used, the result may be better.

### 3.3. Sensitivity at Different Measurement Distances

In this section, whether the measurement distance has an impact on accuracy was studied and a total of three measurement distances (2.50 m, 2.75 m, 3.00 m) were selected. In addition, the measurement was repeated 30 times at each measurement distance. In the experiment, the arrangement of control points and the other settings were the same as in Section 3.2, but the focal length was slightly adjusted with the distance changes. The measured values obtained by the proposed method were compared with the true values of P3HPS, and the average errors and standard deviations of the rotational DOF and the movement DOF of each distance were calculated, as shown in Table 7. The maximum measurement average error of the rotational DOF reached 1.0737° and the minimum measurement average error reached 0.7578°, which appeared at the measurement distances of 3.00 m and 2.50 m, respectively. The maximum and minimum values of the standard deviations appeared at the measurement distances of 3.00 m and 2.75 m, respectively. The maximum and minimum measurement average error of the movement DOF were 0.0067 m and 0.0039 m, respectively, which appeared at the measurement distances of 3.00 m and 2.50 m, and the maximum and minimum values of standard deviations were also found in these two measurement distances. On the whole, the errors of the measurement results of the 2.50 m measurement distance and the degree of dispersion of the data are better than the results of the other measurement distances. Moreover, in the three measurement distances, the accuracy and stability of the measurement results increase as the measurement distance decreases.

**Figure 9:**
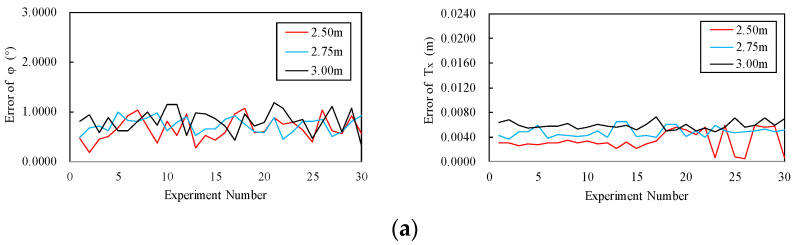

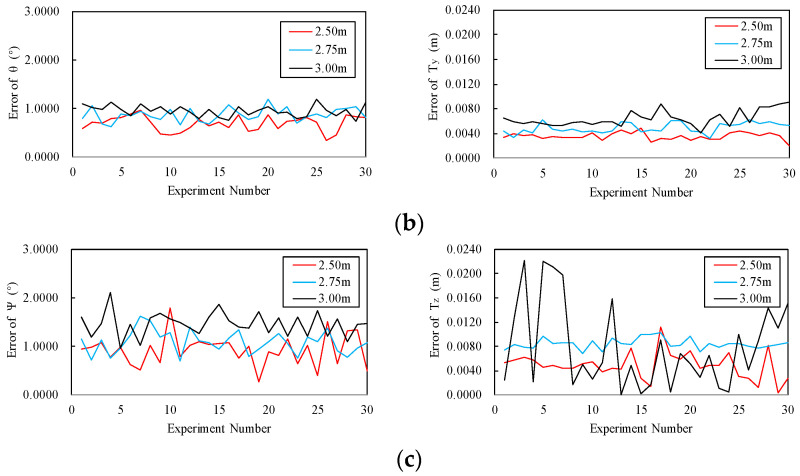
Comparison of measurement errors at different measurement distances: (**a**) measurement errors of the rotational DOF (left) and the movement DOF (right) along the *x*-axis at three measurement distances; (**b**) measurement errors of the rotational DOF (left) and the movement DOF (right) along the *y*-axis at three measurement distances; (**c**) measurement errors of the rotational DOF (left) and the movement DOF (right) along the *z*-axis at three measurement distances.

If the measurement errors of different measurement distances are drawn separately according to different DOF $\left(\varphi ,\;\theta ,\;\Psi ,\;\;T_{x},\;\;T_{y},\;T_{z}\right)$, the line diagrams shown in Figure 9 can be obtained. From the lines in Figure 9a–c, the measurement errors comparison of the three measurement distances can be seen as follows: measurement errors of 2.50 m < measurement errors of 2.75 m < measurement errors of 3.00 m. It can also be seen from the volatility of the lines that the standard deviation of the measurement error of 2.50 m was the smallest, and that of 3.00 m was the largest. This is consistent with the results presented in Table 7. In addition, Figure 9c shows the measurement errors of the rotational DOF and the movement DOF in the *z*-axis direction. Compared with the *x*-axis measurement error lines in Figure 9a and the *y*-axis measurement error lines in Figure 9b, the measurement errors in the *z*-axis direction and the degree of dispersion were slightly larger than those in the *x* and *y* axes directions; this again verified the experimental results in Section 3.2.

It can be seen from Table 7 and Figure 9 that, among the three measurement distances, the measurement results at 2.50 m were more accurate and more stable than the results of the other two measurement distances, but more experiments need to be carried out to find the optimal measurement distance. The reasons for this phenomenon can be summarized in three aspects: pixel coordinates reading, lens distortion, and camera calibration. (1) In terms of pixel coordinates reading, on the basis of the pixel coordinates, it is calculated by Equations (14) and (15) after reading the pixel coordinates of the control points. According to the calculation equation and Figure 4, it is obvious to know that, when there is a certain error in the pixel coordinates, the error of the calculation result of Equation (14) increases as the measurement distance increases. As a result, it may cause the error of the 6-DOF measurement to increase. (2) In terms of lens distortion, the lens assembly will inevitably cause errors, which will cause lens distortion. The distortion of the lens will cause some pixels in the image to shift, so that the pixel coordinates of the control points in the image are inconsistent with the actual situation. As a result, even without reading errors, the pixel coordinates are still inaccurate, and the impact of inaccurate pixel coordinates is the same as the point of view in the previous part. (3) In terms of camera calibration, because the calibration data obtained by camera calibration are used throughout the entire measurement process, the impact it produces is conceivable.

## 4. Conclusions

In this paper, a multi-camera universal measurement method for 6-DOF of rigid bodies in the world coordinate system is proposed. This method only needs to use at least two cameras to achieve measurement, which is made available for most morphological rigid bodies. First of all, on the basis of Zhang Zhengyou’s calibration method, multi-camera calibration is introduced. The pose relationship between the camera coordinate system and the world coordinate system is obtained, which is different from other calibrations. Meanwhile, the intrinsic and extrinsic parameters of the camera are estimated. Secondly, on the basis of the pinhole camera model, the 6-DOF solution model of the proposed method is gradually analyzed by the matrix analysis method. The proposed method uses the control points on the measured rigid body to achieve the calculation of the 6-DOF by least squares methods. Finally, P3HPS (Phantom 3D high-speed photogrammetry system) with an accuracy of 0.1 mm/m was used to evaluate the performance of proposed method. The experiment results show that the average error of the rotational DOF is less than 1.1 deg, and the average error of the movement DOF is less than 0.007 m.

The proposed method does not need expensive and professional instruments. The measurement process is simple and the principle is not complicated. As the final measurement results of the proposed method are the 6-DOF between the world coordinate system and the measured rigid body coordinate system, the measurement is reproducible and the measurement results of the 6-DOF of the rigid body are not restricted by the movement of the camera, which is different from other vision measurement methods. Certainly, the proposed method still has limitations. On the one hand, for a measured rigid body with a too large moving range, it may exceed the camera’s field of view, resulting in an inability to measure. On the other hand, there are more dynamic measurement problems to be solved [48,49,50], which will be the key problem of our research in the future.

## Figures and Tables

**Table 1:** Parameters of the two high-speed cameras.

Camera Model	Maximum Resolution	Sensor Size	Single Pixel Size
Phantom VEO 410	1280 × 800	25.6 × 16.0 mm	20 × 20 μm
Phantom M310			

**Table 2:** Intrinsic parameters of the two cameras.

Camera Model	Principal Point Coordinate (Pixel)	Focal Length (mm)
Phantom VEO 410	(649.47, 389.80)	23.91
Phantom M310	(656.50, 406.79)	23.95

**Table 3:** Rotation matrices from the $C_{1}$ system to the W system.

Image Number	$R_{C_{1}}^{W}=R_{C_{1}}^{S_{l}}$		Standard Deviations (°)		
	Matrix	Vector (°)	*x*	*y*	*z*
1	$\left[\begin{array}{ccc} 0.9975 & 0.0453 & 0.1058\\ 0.0554 & -0.9997 & 0.0001\\ 0.1068 & 0.0076 & -0.9978 \end{array}\right]$	$\left[\begin{array}{ccc} -179.46 & -4.53 & -9.54 \end{array}\right]$	0.2121	0.1183	0.3417
2	$\left[\begin{array}{ccc} 0.9975 & 0.0431 & 0.1074\\ 0.0540 & -0.9996 & -0.0125\\ 0.1081 & 0.0201 & -0.9976 \end{array}\right]$	$\left[\begin{array}{ccc} -178.74 & -4.35 & -9.63 \end{array}\right]$			
3	$\left[\begin{array}{ccc} 0.9970 & 0.0444 & 0.1133\\ 0.0549 & -0.9997 & -0.0061\\ 0.1141 & 0.0139 & -0.9973 \end{array}\right]$	$\left[\begin{array}{ccc} -179.07 & -4.46 & -10.18 \end{array}\right]$			
4	$\left[\begin{array}{ccc} 0.9970 & 0.0439 & 0.1138\\ 0.0541 & -0.9997 & -0.0020\\ 0.1148 & 0.0098 & -0.9973 \end{array}\right]$	$\left[\begin{array}{ccc} -179.30 & -4.40 & -10.25 \end{array}\right]$			
5	$\left[\begin{array}{ccc} 0.9969 & 0.0440 & 0.1148\\ 0.0545 & -0.9997 & -0.0049\\ 0.1156 & 0.0127 & -0.9972 \end{array}\right]$	$\left[\begin{array}{ccc} -179.13 & -4.42 & -10.32 \end{array}\right]$			
6	$\left[\begin{array}{ccc} 0.9977 & 0.0409 & 0.1046\\ 0.0511 & -0.9998 & -0.0032\\ 0.1055 & 0.0106 & -0.9979 \end{array}\right]$	$\left[\begin{array}{ccc} -179.30 & -4.13 & -9.42 \end{array}\right]$			
7	$\left[\begin{array}{ccc} 0.9972 & 0.0446 & 0.1109\\ 0.0549 & -0.9997 & -0.0039\\ 0.1118 & 0.0117 & -0.9975 \end{array}\right]$	$\left[\begin{array}{ccc} -179.21 & -4.47 & -9.98 \end{array}\right]$			
Average	$\left[\begin{array}{ccc} 0.9973 & 0.0437 & 0.1101\\ 0.0541 & -0.9997 & -0.0046\\ 0.1110 & 0.0123 & -0.9975 \end{array}\right]$	$\left[\begin{array}{ccc} -179.17 & -4.93 & -9.90 \end{array}\right]$			

**Table 4:** Translation vectors from the $C_{1}$ system to the W system.

Image Number	$T_{C_{1}/S_{l}}^{S_{l}}(m)$	$T_{W/S_{l}}^{S_{l}}(m)$	$T_{C_{1}/W}^{W}=T_{C_{1}/S_{l}}^{S_{l}}-T_{W/S_{l}}^{S_{l}}(m)$
1	${\left[\begin{array}{ccc} 0.2229 & -0.6169 & 2.0770 \end{array}\right]}^{T}$	${\left[\begin{array}{ccc} 0.510 & -0.686 & -1.600 \end{array}\right]}^{T}$	${\left[\begin{array}{ccc} -0.2871 & 0.0691 & 3.6770 \end{array}\right]}^{T}$
2	${\left[\begin{array}{ccc} 0.2335 & -0.6146 & 2.1757 \end{array}\right]}^{T}$	${\left[\begin{array}{ccc} 0.510 & -0.686 & -1.500 \end{array}\right]}^{T}$	${\left[\begin{array}{ccc} -0.2765 & 0.0714 & 3.6757 \end{array}\right]}^{T}$
3	${\left[\begin{array}{ccc} 0.2413 & -0.6128 & 2.2760 \end{array}\right]}^{T}$	${\left[\begin{array}{ccc} 0.510 & -0.686 & -1.400 \end{array}\right]}^{T}$	${\left[\begin{array}{ccc} -0.2687 & 0.0732 & 3.6760 \end{array}\right]}^{T}$
4	${\left[\begin{array}{ccc} 0.2490 & -0.6113 & 2.3726 \end{array}\right]}^{T}$	${\left[\begin{array}{ccc} 0.510 & -0.686 & -1.300 \end{array}\right]}^{T}$	${\left[\begin{array}{ccc} -0.2610 & 0.0747 & 3.6726 \end{array}\right]}^{T}$
5	${\left[\begin{array}{ccc} 0.2555 & -0.6095 & 2.4713 \end{array}\right]}^{T}$	${\left[\begin{array}{ccc} 0.510 & -0.686 & -1.200 \end{array}\right]}^{T}$	${\left[\begin{array}{ccc} -0.2545 & 0.0765 & 3.6713 \end{array}\right]}^{T}$
6	${\left[\begin{array}{ccc} -0.2471 & -0.6185 & 2.5041 \end{array}\right]}^{T}$	${\left[\begin{array}{ccc} 0.010 & -0.686 & -1.200 \end{array}\right]}^{T}$	${\left[\begin{array}{ccc} -0.2571 & 0.0675 & 3.7041 \end{array}\right]}^{T}$
7	${\left[\begin{array}{ccc} -0.2490 & -0.6202 & 2.4085 \end{array}\right]}^{T}$	${\left[\begin{array}{ccc} 0.010 & -0.686 & -1.300 \end{array}\right]}^{T}$	${\left[\begin{array}{ccc} -0.2590 & 0.0658 & 3.7085 \end{array}\right]}^{T}$

**Table 5:** Calibration comparisons between the proposed method and Phantom 3D high-speed photogrammetry system (P3HPS).

	Multi-Camera Calibration	P3HPS Calibration	Errors		
			*x*	*y*	*z*
$R_{C_{2}}^{C_{1}}$ **(°)**	$\left[\begin{array}{ccc} 1.34 & -1.30 & -2.03 \end{array}\right]$	$\left[\begin{array}{ccc} 1.57 & -1.04 & -1.73 \end{array}\right]$	0.23	0.26	0.30
$T_{C_{2}/C_{1}}^{C_{1}}$ **(m)**	${\left[\begin{array}{ccc} -0.1512 & -0.0039 & 0.0222 \end{array}\right]}^{T}$	${\left[\begin{array}{ccc} -0.1490 & -0.0070 & 0.0190 \end{array}\right]}^{T}$	0.0022	0.0031	0.0032
$R_{C_{1}}^{W}$ **(°)**	$\left[\begin{array}{ccc} -179.17 & -4.39 & -9.90 \end{array}\right]$	$\left[\begin{array}{ccc} -179.17 & -4.82 & -10.43 \end{array}\right]$	0	0.43	0.53
$T_{C_{1}/W}^{W}$ **(m)**	${\left[\begin{array}{ccc} -0.2663 & 0.0712 & 3.6836 \end{array}\right]}^{T}$	${\left[\begin{array}{ccc} -0.2707 & 0.0669 & 3.6797 \end{array}\right]}^{T}$	0.0044	0.0043	0.0039

**Table 6:** Average errors and standard deviations of six-degrees-of-freedom (6-DOF) measurement of rigid body.

Parameters	Average Errors	Standard Deviations
φ (°)	0.7844	0.2351
θ (°)	0.9709	0.1229
Ψ (°)	1.4117	0.2618
$T_{x}$ (m)	0.0057	0.0009
$T_{y}$ (m)	0.0066	0.0014
$T_{z}$ (m)	0.0072	0.0043

**Table 7:** Measurement errors and standard deviations at different measurement distances.

Measurement Distances	Rotational DOF		Movement DOF	
	Average Errors (°)	Standard Deviations (°)	Average Errors (m)	Standard Deviations (m)
2.50 m	0.7578	0.2740	0.0039	0.0017
2.75 m	0.8980	0.2334	0.0060	0.0019
3.00 m	1.0737	0.3457	0.0067	0.0042
